# Bicistronic DNA Vaccines Simultaneously Encoding HIV, HSV and HPV Antigens Promote CD8^+^ T Cell Responses and Protective Immunity

**DOI:** 10.1371/journal.pone.0071322

**Published:** 2013-08-08

**Authors:** Vinicius C. Santana, Mariana O. Diniz, Francisco A. M. O. Cariri, Armando M. Ventura, Edécio Cunha-Neto, Rafael R. Almeida, Marco A. Campos, Graciela K. Lima, Luís C. S. Ferreira

**Affiliations:** 1 Department of Microbiology, Biomedical Sciences Institute, University of São Paulo, São Paulo, Brazil; 2 Laboratory of Clinical Immunology and Allergy-LIM60, Division of Clinical Immunology and Allergy, Department of Medicine, University of São Paulo School of Medicine, São Paulo, Brazil; 3 René Rachou Research Center, Fiocruz, Belo Horizonte, Brazil; Federal University of São Paulo, Brazil

## Abstract

Millions of people worldwide are currently infected with human papillomavirus (HPV), herpes simplex virus (HSV) or human immunodeficiency virus (HIV). For this enormous contingent of people, the search for preventive and therapeutic immunological approaches represents a hope for the eradication of latent infection and/or virus-associated cancer. To date, attempts to develop vaccines against these viruses have been mainly based on a monovalent concept, in which one or more antigens of a virus are incorporated into a vaccine formulation. In the present report, we designed and tested an immunization strategy based on DNA vaccines that simultaneously encode antigens for HIV, HSV and HPV. With this purpose in mind, we tested two bicistronic DNA vaccines (pIRES I and pIRES II) that encode the HPV-16 oncoprotein E7 and the HIV protein p24 both genetically fused to the HSV-1 gD envelope protein. Mice i.m. immunized with the DNA vaccines mounted antigen-specific CD8^+^ T cell responses, including *in vivo* cytotoxic responses, against the three antigens. Under experimental conditions, the vaccines conferred protective immunity against challenges with a vaccinia virus expressing the HIV-derived protein Gag, an HSV-1 virus strain and implantation of tumor cells expressing the HPV-16 oncoproteins. Altogether, our results show that the concept of a trivalent HIV, HSV, and HPV vaccine capable to induce CD8^+^ T cell-dependent responses is feasible and may aid in the development of preventive and/or therapeutic approaches for the control of diseases associated with these viruses.

## Introduction

The diseases caused by human immunodeficiency virus (HIV), human papillomavirus (HPV) and herpes simplex virus (HSV) represent serious public health threats, as they affect millions of people irrespective of economic or social status [1]. The mortality and morbidity associated with HIV or HSV infection were significantly reduced after the discovery and dissemination of anti-viral therapies that reduce viral loads and relieve symptoms in infected people. However, the currently available drugs are not able to eradicate the viruses, and infections with these viruses remain in a chronic latent state and recur after treatment interruption (HIV) or after debilitation of the immune defenses (HSV). Despite decades of intense scientific work and enormous investments, no effective anti-HIV or anti-HSV vaccine is presently available [2]. Regarding HPV, two prophylactic vaccines that are able to induce antibody responses have been shown to confer protection against virus infection and therefore reduce the long-term incidence of HPV-associated tumors [3], [4]. However, the impact on the incidence of HPV-associated cancers is expected to be observed only after the widespread use of these vaccines. Nonetheless, those already infected with high-risk HPV types or afflicted with HPV-associated cancer or neoplastic lesions are not expected to benefit from preventive anti-viral vaccines. Therefore, the development of therapeutic cancer vaccines that target HPV-infected cells is a priority for several research groups [5].

The concept of therapeutic vaccines relies on the fact that the activation of immunological mechanisms leading to cytotoxic responses, particularly antigen-specific CD8^+^ T cell activation, permanently eradicates virus-infected or tumor cells [6]. Although theoretically sound and technologically feasible, the development of vaccines that efficiently activate antigen-specific CD8^+^ T cell populations to control the replication of viruses, such as HIV, remains elusive, as dramatically illustrated by the STEP program [7]. Similarly, numerous attempts to develop both prophylactic and therapeutic anti-HSV vaccines have systematically failed, and new insights regarding the immunological control of HSV-1 and HSV-2 infections are eagerly awaited [8], [9]. Vaccines targeting the tumors induced by HPV, under both experimental and clinical conditions, stand as the best and most promising examples of the viability of therapeutic vaccines as immunological tools for the control of infectious and degenerative diseases [10]–[15].

DNA vaccines have been widely used as therapies against tumors and viruses because of their capability to induce antigen-specific CD8^+^ T cell responses as well as their rather simple manipulation [16], [17]. DNA vaccines are also amenable to the development of multivalent formulations either by a mixture of plasmids encoding single antigens or by multiple antigens expressed as fused epitopes [18] or proteins derived from the same or different pathogens [19]–[21]. Multivalent DNA vectors can also be engineered to encode polycistronic transcripts under the control of a single promoter, leading to the simultaneous expression of multiple antigens in transfected host cells [22]–[26].

We have previously shown that DNA vaccines encoding the HPV-16 E7 oncoprotein genetically fused to HSV-1 glycoprotein D (gD) enhance both the induction of E7-specific CD8^+^ T cell responses and therapeutic/prophylactic anti-tumor effects compared to vaccines encoding the non-fused HPV oncoproteins in mice [15], [27], [28]. Additional evidence has indicated that these gD-dependent immunological effects, particularly the activation of CD8^+^ T cell responses to bystander antigens, involve the binding of gD to herpes virus entry mediator (HVEM) and the blockage of a co-inhibitory immune mechanism involving the B- and T-lymphocyte attenuator (BTLA) cell receptor [13], [29]. In addition, further experimental evidence has indicated that the binding of gD to HVEM not only represents an important step in virus entry but also triggers an NF-kB-activation pathway and inhibits apoptosis in HVEM-expressing cells, such as antigen-presenting cells [30], [31].

More recently, the fusion of gD with the influenza virus nucleoprotein further demonstrated that an adjuvant effect on CD8^+^ T cell responses can be achieved with different antigens and reveals the possibility of the development of multivalent anti-virus vaccine formulations that are able to induce prophylactic and/or therapeutic protective immune responses [32]. In the present study, we applied this vaccine technology to develop trivalent DNA vaccines encoding the HSV-1 protein gD genetically fused with both the HPV-16 oncoprotein E7 and the HIV-1 protein p24 by the same transfected cell. To achieve the simultaneous expression of the two hybrid proteins using a single vaccine vector, we employed a bicistronic expression system using an internal ribosome entry site (IRES) sequence [23], [24]. Two vaccine vectors were constructed, pIRES I and pIRES II, which expressed the recombinant hybrid proteins under the control of a strong virus promoter (CMV) but differed in the order of the cistrons with respect to the promoter. Mammalian cells transfected with the recombinant vectors simultaneously expressed the HSV, HIV and HPV proteins at the cell surface. More importantly, C57BL/6 or BALB/c mice immunized with the tested vaccines elicited antigen-specific CD8^+^ T cell responses and were protected to challenges with a vaccinia virus expressing the HIV-1 protein Gag and a HSV-1 strain. In addition, vaccine administration could therapeutically eradicate the growth of tumor cells expressing the HPV-16 oncoproteins implanted in C57BL/6 mice. Altogether, the present study demonstrates that the generation of a trivalent HIV, HSV and HPV anti-cancer vaccine is a feasible goal and should be pursued as a potential tool to prevent and/or treat infections with these viruses.

## Results

### Generation of Trivalent DNA Vaccines that Simultaneously Encode HIV, HSV and HPV Antigens

**Figure 1:**
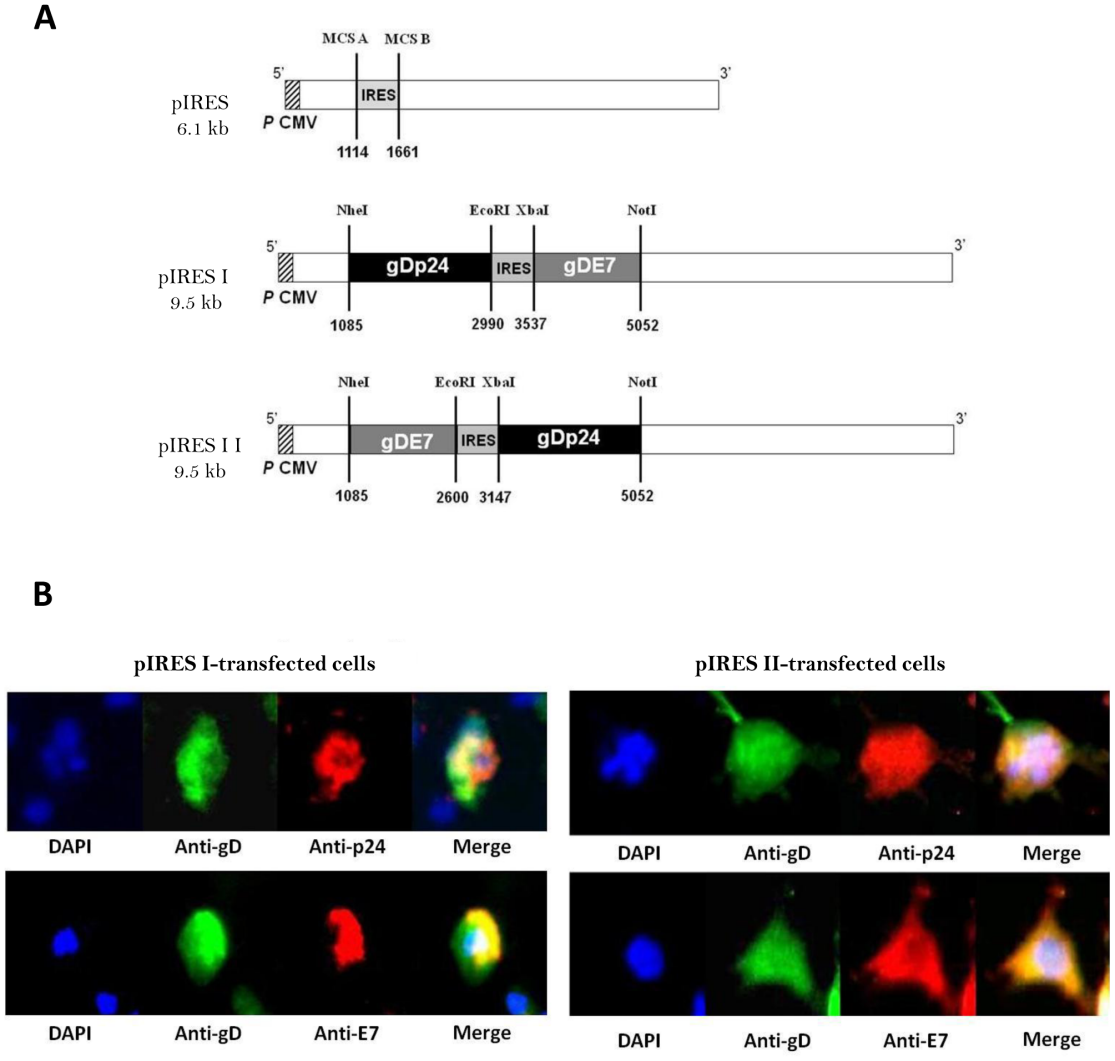
Construction of bicistronic DNA vaccines encoding HPV, HIV and HSV antigens for expression in mammalian cells. (A) Schematic linear representation of the trivalent DNA vaccines. pIRES I and pIRES II contain gDp24 and gDE7 chimeric gene fusions, which are inverted with regard to the CMV promoter and IRES sequence. The empty vector pIRES Ø was used as a control. The nucleotide numbers corresponding to the IRES sequence and the cloned chimeric genes are indicated. (B) In vitro expression of the chimeric proteins encoded by pIRES I (left panels) and pIRES II (right panels). Non-permeabilized pIRES I- or pIRES II-transfected COS-7 cells were labeled with antigen-specific antibodies for the simultaneous detection of the HSV-1 protein gD and the HIV-1 protein p24 or the HPV-16 oncoprotein E7. Green, gD; red, p24 or E7; yellow, co-localization of gD with p24 or E7; blue, DAPI nuclear staining.

The DNA vaccines tested in this study were obtained by amplifying the chimeric HPV-16 E7 or HIV-1 p24 coding sequences, both of which were fused to the HSV-1 gD sequence, and cloning them into the pIRES vector. This process generated two bifunctional plasmids, pIRES I and pIRES II, which only differ in the order of their cloned chimeric genes (**Figure 1A**). pIRES I carries the gDp24 gene fusion upstream of the IRES sequence and the gDE7 gene fusion downstream of this regulatory sequence. In contrast, the pIRES II vector carries the gDE7 and gDp24 sequences upstream and downstream of the IRES sequence, respectively. To confirm the expression and cellular localization of the encoded hybrid proteins, the recombinant DNA vaccines were introduced into mammalian COS-7 cells, and the encoded proteins were observed by immunofluorescence. The HSV-1 protein gD is a viral envelope glycoprotein that is expressed on the surface of infected cells via a C-terminal anchor sequence [33]. Thus, the recombinant proteins were detected in non-permeabilized cells treated with specific monoclonal (gD) or polyclonal (p24 and E7) antibodies against the target antigens. Cells transfected with pIRES I or pIRES II were simultaneously stained with antibodies against all three viral antigens (**Figure 1B**). Signals corresponding to the three proteins were detected mainly at the surface of the non-permeabilized cells. These results indicate that the hybrid proteins were correctly folded and properly targeted to the cell membrane compartment of the pIRES I- and pIRES II-transfected cells.

### pIRES I and pIRES II Vaccination Induces the Activation of Antigen-specific CD8^+^ T Cells and Simultaneous Protective Responses against Gag-expressing Vaccinia Virus and Tumor Cells Expressing HPV-16 Antigens

**Figure 2:**
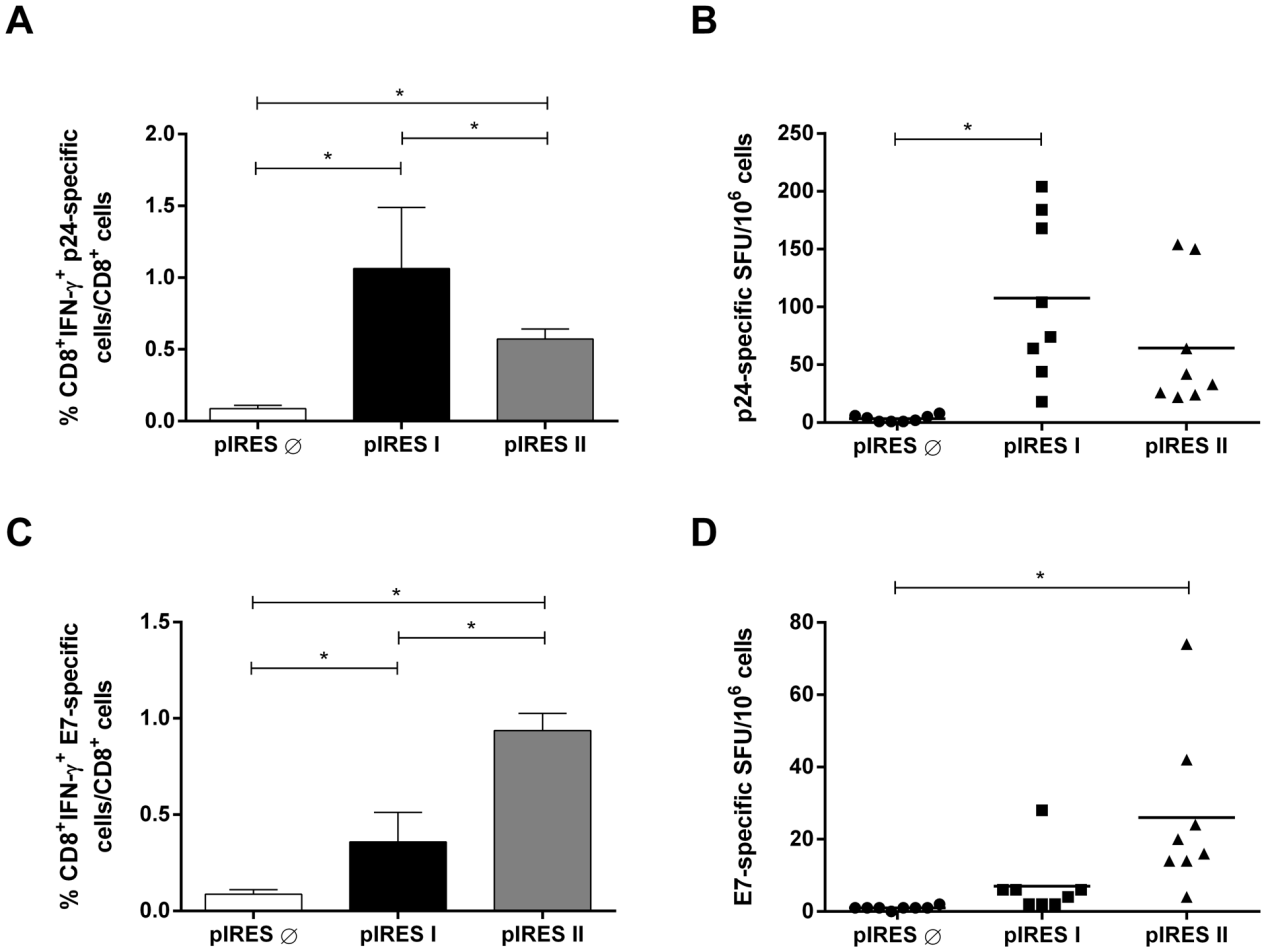
Activation of antigen-specific IFN-γ-producing CD8+ T cell precursors in mice immunized with pIRES I or pIRES II. (A-B) Spleen cells from BALB/c mice spleen cells were stimulated with the MHC-I-restricted p24-specific peptide, and the p24-specific IFN-γ-producing CD8^+^ T cells were detected by intracellular cytokine staining (A) or ELISPOT assay (B). (C–D) Spleen cells from C57BL/6 mice were stimulated with the MHC-I-restricted E7-specific peptide, and the E7-specific IFN-γ-producing CD8^+^ T cells were detected by IFN-γ intracellular staining (C) or ELISPOT assay (D). Mice were i.m. immunized with three doses of the DNA vaccines with one week intervals between doses (100 µg/dose). The CD8^+^ T-cell responses were analyzed two weeks after the last dose. *p<0.05. Data represent the compilation of two independent experiments with four mice per immunization group (n = 8) and results expressed by each animal analyzed. pIRES is the empty vector used as immunization control.

Mice were immunized with pIRES I and pIRES II and monitored for the activation of E7-specific (in C57BL/6 mice) and p24-specific (in BALB/c mice) CD8^+^ T cells, the key immunological response required for both the control of virus-induced tumors and intracellularly replicating viruses. The CD8^+^ T cell mediated responses were first monitored in the vaccinated mice by detecting the level of antigen-specific IFN-γ^+^ -producing CD8^+^ T cells using both ICS and ELISPOT assays for total spleen cells harvested fourteen days after the last immunization dose. The cells harvested from the vaccinated mice were incubated with synthetic peptides corresponding to the immunodominant HPV-16 E7 and HIV-1 p24 MHC-I-restricted CD8^+^ T cell-specific epitopes for the H-2K^b^ and H-2K^d^ haplotypes, respectively. Cells stimulated with the H-2K^d^-restricted p24 peptide for 6 h resulted in the activation of p24-specific IFN-γ^+^/CD8^+^ T cells at frequencies of approximately 1% and 0.6% in BALB/c mice immunized with pIRES I and pIRES II, respectively, as determined by ICS (**Figure 2A**). Following the same stimulation procedure with the p24-specific peptide but evaluating the results with an ELISPOT assay, only the animals vaccinated with pIRES I reached a statistically higher response compared with the control group immunized with the empty DNA vector (**Figure 2B**). The E7-specific IFN-γ^+^/CD8^+^ T cell responses elicited in the vaccinated C57BL/6 mice were determined using the H-2K^b^-restricted E7-specific peptide. Intracellular IFN-γ staining showed that the animals immunized with pIRES I or pIRES II developed higher E7-specific IFN-γ^+^/CD8^+^ cell responses compared with mice immunized with the control vector, while only the animals immunized with the pIRES II vector generated positive responses compared with the control group when the responses were analyzed with the ELISPOT assay (**Figure 2C and D**). In addition, the E7-specific responses detected in the mice immunized with pIRES II were statistically higher than those detected in the mice immunized with pIRES I, as monitored by ICS (**Figure 2C**). Collectively, these results indicate that immunization with pIRES I and pIRES II induces p24- and E7-specific CD8^+^ T cell responses in mice, but higher E7-specific IFN-γ^+^/CD8^+^ T cell responses are present in mice immunized with the vector in which the E7/gD fusion is encoded by the first cistron (pIRES II).

**Figure 3:**
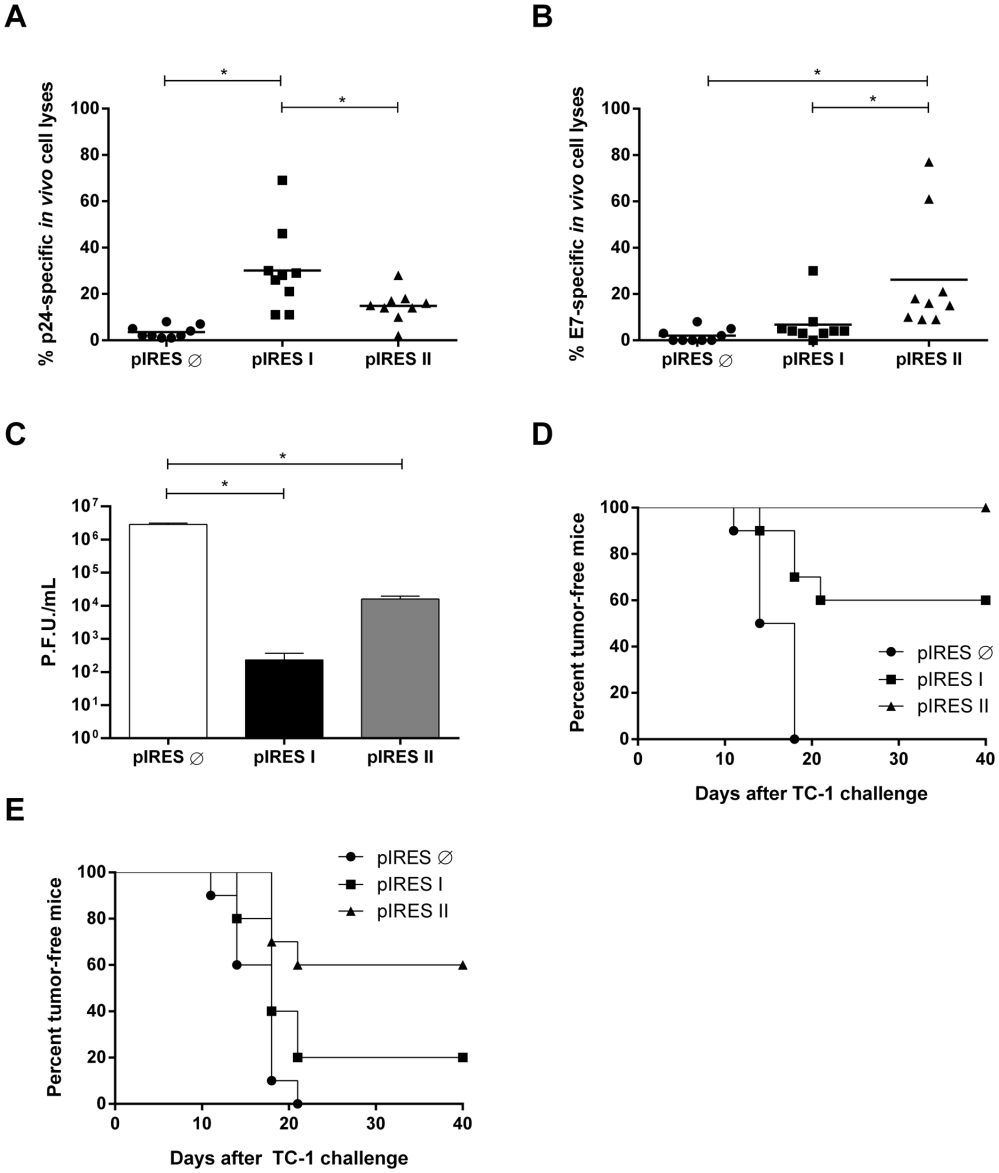
Induction of *in vivo* E7- and p24-specific cytolytic CD8^+^ T cell responses in immunized mice. (A–B) *In vivo* antigen-specific CD8^+^ T cell-dependent cytotoxic responses in the vaccinated mice was measured two weeks after the last immunization dose. Spleen cells from BALB/c or C57BL/6 mice were labeled with CFSE and pulsed with synthetic peptides representing the immunodominant MHC-I-restricted epitopes of p24 (A) or E7 (B). Data shown in A and B represent the compilation of two independent experiments, encompassing four and five mice per group (n = 9) with results based on the response of each animal. (C) The protective immunity elicited in BALB/c mice immunized with pIRES I or pIRES II was measured after challenge with a recombinant vaccinia virus expressing the HIV-1 protein Gag. Female BALB/c mice were challenged with 2×10^6^ P.F.U. of rVV-Gag, and 5 days later, the level of viable vaccinia virus in the ovaries was determined after titration in Vero cells. Data shown in C represent the compilation of two independent experiments carried out with pooled samples from five mice per group. *p*<*0.05. (D) Prophylactic and (E) therapeutic anti-tumor immunity in C57BL/6 mice immunized with pIRES I or PIRES II. The prophylactic anti-tumor effects were determined in five vaccinated female mice after the s.c. transplantation of 7.5×10^4 ^TC-1 cells two weeks after the last vaccination. The therapeutic anti-tumor effects induced by the vaccines were determined after transplantation of 7.5×10^4 ^TC-1 cells one day before the administration of the first vaccine dose. Data shown in D and E represent the compilation of two independent experiments, with five mice per group. The survival curves D and E raised *p* values of 0.0001 and 0.0006, respectively, in the Logrank test for trend. pIRES is the empty vector used as immunization control.

To further demonstrate that the antigen-specific activation of CD8^+^ T cells was functional in the vaccinated mice, we measured the *in vivo* antigen-specific cytotoxic responses elicited in mice immunized with pIRES I or pIRES II. Splenocytes harvested from non-vaccinated mice were surface labeled with both a fluorescent label (CFSE) and either the MHC-I restricted p24- or E7-specific peptide. The labeled cells were intravenously introduced into vaccinated mice, which were monitored 24 h later for the specific lysis of the labeled cells in their spleens. The relative reduction in the level of peptide-labeled cells compared with cells labeled only with CFSE was indicative of antigen-specific CD8^+^ T cell-dependent cytolytic activity. Mice immunized with pIRES I mounted a p24-specific CD8^+^ T cell-dependent cytotoxic response, while mice immunized with pIRES II showed a statistically significant E7-specific CD8^+^ T cell-dependent cytotoxic response compared with mice immunized with the empty vector (**Figure 3A and B**). These results further demonstrate that effective antigen-specific CD8^+^ T cell-dependent cytotoxic responses are induced by the vaccines in which the target gene is expressed by the first cistron of the bicistronic transcript.

As a final step to evaluate the protective immunological status of the mice immunized with the DNA vaccines encoding the HPV and HIV antigens, we submitted the vaccinated mice to challenges with a recombinant vaccinia virus expressing the Gag protein or TC-1 tumor cells expressing the HPV-16 E6 and E7 oncoproteins. As shown in **Figure 3C**, female BALB/c mice immunized with pIRES I or pIRES II showed significantly less of the recombinant vaccinia virus in their ovaries after challenge with the vAbT141 vaccinia strain (1,000- or 100-fold less compared with the control group immunized with the empty DNA vector, respectively). Similarly, C57BL/6 mice immunized with pIRES I or pIRES II developed protective anti-tumor immunity. All mice challenged two weeks after immunization with pIRES II remained tumor free, while 60% of the mice immunized with pIRES I remained tumor-free after transplantation of the TC-1 cells (**Figure 3D**). Notably, mice immunized with the DNA vaccines developed partial therapeutic anti-tumor immunity. Twenty percent of C57BL/6 mice transplanted with TC-1 cells and subsequently treated with three doses pIRES I remained tumor-free, while 60% of the mice immunized with pIRES II developed a therapeutic anti-tumor response, as they were able to eradicate the TC-1 cells that were previously transplanted into their bodies (**Figure 3E**). Collectively, these results indicate that the DNA vaccines are able to induce functional and protective CD8^+^ T cell-dependent responses in vaccinated mice, leading to efficient anti-virus (HIV) and anti-tumor (HPV) effects. In addition, these results indicate that vaccination with these DNA vectors, particularly pIRES II, confers therapeutic immune responses to tumor cells.

### Vaccination with pIRES I or pIRES II Induces gD-specific T Cell Responses and Resistance to HSV-1 Challenges

**Figure 4:**
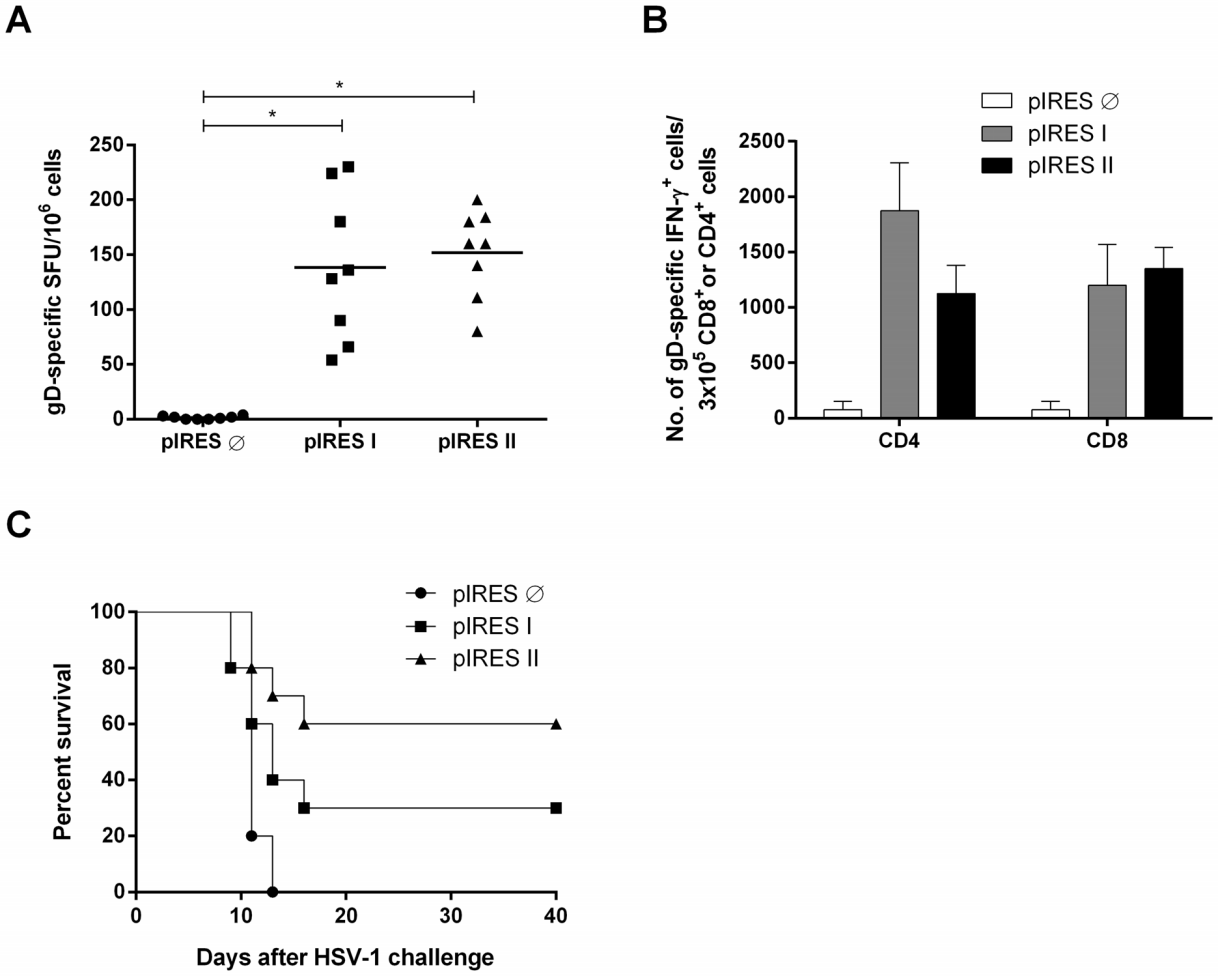
Induction of gD-specific functional T cell responses in mice immunized with pIRES I or pIRES II. (A) Detection of gD-specific IFN-γ-secreting cells in vaccinated BALB/c mice was performed two weeks after the last immunization dose. Spleen cells from individual mice (n = 8) were cultured in the presence of full-length recombinant purified gD for 72 h. The frequencies of the gD-specific IFN-γ-secreting cells were measured by ELISPOT. (B) Detection of gD-specific IFN-γ-secreting CD8^+^ and CD4^+^ T cells in those mice described in (A) with spleen cells previously incubated for 72 h with the recombinant gD analyzed by flow cytometry. Data shown in A and B represent the compilation of two independent experiments, with four mice per group (n = 8). *p<0.05. (C) Protective anti-HSV immunity elicited in mice immunized with pIRES I or pIRES II. Vaccinated male BALB/c mice were challenged intranasal with the HSV-1 strain EK (5×10^4 ^P.F.U./mouse) two weeks after last vaccine dose and mice survival was monitored for 40 days. Data shown in C represent the compilation of two independent experiments, with five mice per group (n = 10). *p* = 0.0009 (Logrank test for trend). pIRES is the empty vector used as immunization control.

The trivalent nature of the immune responses elicited in the mice immunized with pIRES I or pIRES II was confirmed after determination of the HSV gD-specific T cell responses. Two weeks after the last immunization dose, BALB/c mice were monitored for the induction of gD-specific CD4^+^ and CD8^+^ T cell responses using ELISPOT assays. As shown in **Figure 4A**, mice vaccinated with pIRES I or pIRES II developed similar antigen-specific cellular responses, as measured by the number of IFN-γ-secreting cells after stimulation with purified gD, compared with mice immunized with the empty vector. Analysis of the activated antigen-specific T cell populations by flow cytometry indicated that both pIRES I and pIRES II activated the CD4^+^ and CD8^+^ T cells. Nonetheless, the mice immunized with pIRES II activated a higher proportion of gD-specific IFN-γ producing CD8^+^ T cells (CD8^+^/CD4^+^ ratio of 1.2) than mice immunized with pIRES I (CD8^+^/CD4^+^ ratio of 0.64) (**Figure 4B**). pIRES I- and pIRES II-vaccinated mice were also partially protected against lethal challenge with HSV-1. Sixty percent of the BALB/c mice immunized with pIRES II survived the lethal challenge carried out with HSV-1 EK strains, while a lower level of protection (30%) was achieved in mice immunized with pIRES I (**Figure 4C**). In conclusion, these results also confirm that the tested DNA vaccines induce T cell-dependent immune responses and protective immunity against HSV.

## Materials and Methods

### Ethics Statement

All animal handling and immunization procedures were approved by the institutional ethics committee for animal experimentation and care “*CEUA ICB-USP – Comissão de Ética no Uso de Animais do Instituto de Ciências Biomédicas da Universidade de São Paulo*” (protocol number: 12/05/2010-007-82-2) and followed standard rules approved by the Brazilian College of Animal Experimentation (COBEA).

### Mice, Cell Lines and Antibodies

C57BL/6 and BALB/c mice (6–8 weeks old) were provided by the animal facility of the Department of Parasitology at the University of São Paulo. The TC-1 tumor cell lineage [34], which is derived from C57BL/6 mouse lung epithelial cells transformed with the v-Ha-ras oncogene and HPV-16 E6 and E7, was kindly provided by Dr T. C. Wu at John Hopkins University. TC-1 and COS-7 (ATCC® CRL-1651™) cells were cultured in Dulbeccós modified Eagle’s medium (DMEM) supplemented with 10% fetal bovine serum (FBS) and 50 units/mL penicillin/streptomycin and kept at 37°C and 5% CO_2_. For the tumor challenge experiments, TC-1 cells were harvested after trypsinization, washed twice, and suspended in serum-free media at the appropriate concentrations for injection. Rabbit anti-gD, mouse anti-p24 and mouse anti-E7 polyclonal antibodies were generated in our laboratory by subcutaneous injection of four doses of the specific recombinant proteins in combination with alum.

### Cloning of gDp24 and gDE7 in the pIRES Vector

The gDE7 and gDp24 gene fusions were obtained by PCR amplification using the plasmids pgDE7 [27] and pgDp24 as templates, which contain the coding sequences of HPV-16 E7 and HIV-1 clade B p24, respectively, each fused to the coding sequence of HSV-1 gD. The inserts were amplified using the primers gDFwXbaI (5′ TAG TCT AGA ATG GGG GGG GCT GCC GCC AGG 3′, *XbaI* restriction site underlined) and gDFwNheI (5′ TAG GCT AGC ATG GGG GGG GCT GCC GCC AGG 3′, *NheI* restriction site underlined) for subcloning upstream of the IRES or the primers gDRvNotI (5′ TAG GCG GCC GCG CAC CCA TTA AGG GGG GGT ATC 3′, *NotI* restriction site underlined) and gDRvEcoRI (5′ TAG GAA TTC GCA CCC ATT AAG GGG GGG TAT C 3′, *EcoRI* restriction site underlined) for subcloning downstream of the IRES. The amplicons were cloned into the pGEM™ cloning vector and then double digested with *NheI/EcoRI* or *XbaI/NotI* (Invitrogen, Carlsbad, CA). The resulting fragments were subcloned into the pIRES™ vector (Clontech, Mountain View, CA). The plasmid containing the gDp24 gene fusion upstream of the IRES and gDE7 downstream of the IRES was named pIRES I. The plasmid containing the gDE7 gene fusion upstream of the IRES and gDp24 downstream of the IRES was named pIRES II. All plasmid constructs were confirmed by automatic DNA sequencing. The final vectors were propagated in *Escherichia coli* DH5-α in LB medium supplemented with ampicillin (100 µg/mL) and purified using a Qiagen Plasmid Mega Kit (Qiagen, Hilden, Germany).

### Cell Transfection and Immunofluorescence Analysis

COS-7 cells were cultivated in 12-well culture plates at an initial concentration of 2×10^5^ cells/well in DMEM supplemented with 10% FBS. On the following day, the cells were transfected using Lipofectamine™ 2000 (Invitrogen) according to the manufactureŕs instructions. After 36 h, the cells were harvested with PBS containing 2 mM EDTA, pelleted and incubated with blocking solution (1% bovine serum albumin in PBS) for 20 min at room temperature. The cells were washed twice with PBS and then incubated for 45 min with a 1:100 dilution of a rabbit specific anti-gD serum and a mouse specific anti-E7 serum or the same anti-gD serum combined with a mouse specific anti-p24 serum. The cells were washed twice with PBS and then incubated for 45 min with a 1:200 dilution of FITC-labeled goat anti-mouse IgG (Sigma) or a 1:200 dilution of Texas Red-labeled goat anti-rabbit IgG (Sigma). The cells were washed twice with PBS, stained with a 1:1,000 dilution of DAPI (1 mg/mL) and visualized for immunofluorescence using an inverted microscope (Axiovert model S100, Zeiss) equipped with a digital camera (Hamamatsu model C5810, Sanyo Denki).

### DNA Vaccination and *in vivo* Protection Experiments

BALB/c or C57BL/6 mice were intramuscularly (i.m.) immunized with 3 doses of the DNA vaccines at weekly intervals, with 100 µg DNA/dose in PBS in a final volume of 100 µL. Tumor growth prevention was determined after subcutaneous transplantation of 7.5×10^4 ^TC-1 cells/mouse in the right flank two weeks after the last immunization. Therapeutic anti-tumor effects were measured after transplantation of the TC-1 cells and administration of ththe first vaccine dose one day later. Tumor growth was monitored by visual inspection and palpation twice per week over an observation period of 60 days. The prophylactic anti-HSV effects in the vaccinated BALB/c mice were determined using the HSV-1 EK strain (kindly provided by Dr. Erna Geessien Kroon of the Universidade Federal de Minas Gerais), which was diluted in PBS, and 10-µL aliquots (containing 5×10^4 ^P.F.U./dose) were delivered in the mouse nostrils 15 days after the last immunization dose. The challenged mice were monitored for survival for 40 days.

To evaluate the prophylactic anti-HIV immune response induced by the vaccines, mice were challenged with recombinant vaccinia virus expressing the HIV-1 protein Gag (rVV-Gag), which was supplied by the NIH (vaccinia strain vABT-141). Challenge experiments were performed by i.p. inoculating female BALB/c mice with 2×10^6 ^P.F.U. of rVV-Gag in 100 µL of saline two weeks after the last immunization. The mice were euthanized, and their ovaries were harvested and macerated with a syringe plunger in 1 mL DMEM. The suspensions were submitted to 2-fold serial dilution, 100 µL of each dilution was incubated with Vero cells for 1 h, and the medium was replaced. After 72 h, the cells were stained with a solution containing 20% alcohol and 0.2% of violet crystal, and subsequently inspected for the viral plaques.

### Determination of CD8+/IFN-γ^+^ T Cell Response by Intracellular Cytokine Staining (ICS) and ELISPOT Assays

Spleen cells were harvested two weeks after the last immunization dose, suspended in RPMI 1640 supplemented with 5% SFB and treated with ACK lysing buffer for 5 min on ice to eliminate the red blood cells. For intracellular IFN-γ staining, spleen cells (3×10^6^ cells/well) were cultured at 37°C in 96-well round-bottom microtiter plates (BD Biosciences) in 200 µL of RPMI 1640 medium supplemented with penicillin (100 U/mL), streptomycin (100 µg/mL), 2-ME (50 mM), L-glutamine (2 mM), sodium pyruvate (1 mM), 10% heat-inactivated FBS and brefeldin A (GolgiPlug, BD Biosciences) at a final concentration of 10 µg/mL. In vitro stimulation was achieved using the MHC-I-restricted (H-2K^b^) E7-specific peptide (E7_49–57_; RAHYNIVTF) or the MHC-I-restricted (H-2K^d^) p24-specific peptide (gag_197–205_; AMQMLKETI) at a concentration of 3 µg/mL for 6 h or recombinant gD at a concentration of 10 µg/mL for 72 h (brefeldin A was added in the last 6 h of culture). The cells were incubated for 30 min at 4°C with 100 µL of a 1:100 dilution of FITC-conjugated mAb anti-mouse CD8a and Pe-Cy5-conjugated mAb anti-mouse CD4 (BD Biosciences), washed with PBS containing 2% FBS, fixed with 4% paraformaldehyde for 20 min and then permeabilized with 0.5% saponin for 20 min at 4°C. After washing, the cells were incubated for 30 min at 4°C with a 1:100 dilution of PE-labeled mAb anti-mouse IFN-γ (BD Biosciences). The cells were examined with a FACSCalibur™ cytometer (BD Biosciences), and the data were analyzed using FlowJo software (Tree Star). ELISPOT assays were carried out after the incubation of splenocytes with the same stimuli used in the ICS assay but for 24 hours for peptide stimulation and 72 hours for protein stimulation. Spleen cells (5×10^5^/well) were seeded for in vitro stimulation in nitrocellulose-bottom plates that were previously coated with anti-IFN-γ. IFN-γ secretion was detected after staining with a biotinylated anti-IFN-γ antibody overnight at 4°C and treatment with the AEC Substrate Set (BD Biosciences) according to manufacturer’s instructions.

### *In vivo* Antigen-specific Cytotoxicity Assays

Naïve mouse splenocytes were stained with 0.5 µM or 5 µM of carboxy fluorescein diacetate succinimidyl ester (CFSE) (Invitrogen). The cells labeled with 5 µM CFSE were pulsed with 2.5 µg/mL of the E7-specific peptide (RAHYNIVTF) or the p24-specific (AMQMLKETI) peptide for 40 min at 37°C. Equal amounts of both cell populations (2×10^7^ cells) were intravenously (i.v.) inoculated into C57BL/6 or BALB/c mice, for determination of anti-E7 or anti-p24 responses, respectively, that were previously submitted to the immunization regimens two weeks after the last immunization dose. One day later, the spleens of the inoculated mice were harvested and monitored by flow cytometry. The percentage of target cell killing by specific cytotoxic CD8^+^ T lymphocytes (CTL) was determined as previously described [35].

### Statistical Analyses

All data, which are expressed as means ± SD, are representative of at least five mice per group, and all experiments were repeated twice. One-way ANOVA followed by Bonferroni’s post-test was employed whenever individual data points were compared. The survival and tumor development curves were submitted to log-rank tests. Differences with p≤0.05 were considered to be statistically significant.

## Discussion

The three most relevant viral pathogens associated with sexually transmitted diseases, HIV, HSV and HPV, share common features, such as mucosal transmission, the ability to establish chronic infections, and the requirement of antigen-specific cytotoxic CD8^+^ T cell activation for the efficient control of intracellular virus replication. So far the search for vaccines against these pathogens has emphasized strategies involving the induction of antibodies that are able to neutralize the viruses. Indeed, such approaches have been successful, as two preventive and lucrative anti-HPV vaccines have been generated and are currently being used in several countries [3], [4]. Nonetheless, there is a strong belief that the immunological control of HIV and HSV as well as the tumors induced by HPV, either prophylactic or therapeutically, will require the implementation of alternative methodological tools. In the present study, we propose a trivalent vaccine approach in which bicistronic DNA vaccines simultaneously encoding antigens of HIV, HSV and HPV activate antigen-specific and functionally active CD8^+^ T cell responses under experimental conditions. Moreover, our study demonstrates that the tested vaccine formulations confer protective immunity to all three antigens from HIV, HPV and HSV viruses, as observed in mice challenged with an HSV-1 strain or a recombinant vaccinia virus encoding the HIV-1 protein Gag and in mice implanted with tumor cells expressing the HPV-16 oncoproteins. Altogether, our results indicate that the development of multivalent vaccines inducing protective immunity against HIV, HSV and HPV are feasible and further support the relevance of cytotoxic T cell responses for the protective immunity to these pathogens.

Our proposed vaccine strategy is based on two methodological approaches that induce protective immunity. First, the use of bicistronic DNA vectors that allow for the simultaneous expression of two chimeric proteins (HIV-1 p24 and HPV-16 E7 that are both genetically fused to the HSV-1 envelope protein) gD in the same transfected cells permits a more balanced and efficient induction of CD8^+^ T cell responses in vaccinated subjects. Second, chimeric proteins in which both the HIV-1 protein p24 and the HPV-16 oncoprotein E7 are fused to the HSV-1 protein gD at its C-terminus are targeted to the surface of the transfected cells. In particular, this second feature results in the induction of gD-specific immunomodulatory pathways and the subsequent enhanced activation of CD8^+^ T cell responses to the encoded antigens and, therefore, to the protective immunity generated in the vaccinated animals [15], [32], [36]. The rationale behind the use of DNA vaccine vectors is well established and includes the potential to activate cellular immune responses without the use of exogenous adjuvants, the lack of interference from previously established immunity, an excellent safety record and a rather simple and low-cost production pipeline [37]. Characteristically, DNA vectors, like most eukaryotic genes, are monocistronic, with a single structural gene under the control of a promoter. The discovery of IRES sequences revealed the possibility of generating polycistronic DNA vectors, leading to the simultaneous activation of immune responses to different antigens without the need to transfect cells several plasmids [23], [24], [38], [39]. Our results confirmed that both of our DNA vectors constructed with this IRES technology promoted the synthesis of the target antigens in the same transfected cell. In addition, immunization of mice with pIRES I or pIRES II elicited antigen-specific CD8^+^ T cell responses and *in vivo* cytotoxic responses. These results may reflect the differential expression of the antigens encoded by the two cistrons, with higher expression for the proximal genes compared with the distal cistron. Similar effects have been previously reported by other groups [24], [25], [40], and most likely reflect premature transcript termination at the IRES sequence, which reduces the level of full-length mRNA and, consequently, the amount of the protein encoded by the second cistron. Induction of a more balanced immune response to the target antigens could, thus, be achieved after the concomitant administration of both pIRES I and II vectors. Nonetheless, the present results indicate that the bicistronic expression technology still needs further improvement in order to be successfully used at in vivo situations, particularly, as vaccines.

The efficient activation of T cell responses involves the correct processing and surface presentation of epitopes by APCs and the correct signaling between APCs and effector T cells, which is mediated by surface-exposed receptors, by means of different co-stimulatory and co-inhibitory pathways [41], [42]. The HSV-1 protein gD encoded by our DNA vaccines plays dual roles in the induction of protective anti-viral responses. First, it acts as a highly conserved protective antigen for both the HSV-1 and HSV-2 strains, and second, it exerts strong and complex immunomodulatory effects, enhancing the activation of the CD8^+^ T cell response to its genetically fused antigens [29], [43]. The entry of both HSV-1 and HSV-2 into the host cell is mediated, at least in part, by the specific interaction between gD and HVEM, a member of the TNFR family [44]. In addition, the binding of gD to HVEM blocks the co-inhibitory pathway by competing with BTLA and CD160 [43]. In contrast, the binding of gD does not interfere with the co-stimulatory functions of HVEM, which involve other surface proteins, such as LIGHT and lymphotoxin-α, and lead to the stimulation of the transcription factors NF-kβ and AP-1 and the subsequent activation and differentiation of effector T cells [45], [46]. Further experimental evidence has demonstrated that the binding of gD to HVEM activates the NF-kβ-dependent signaling pathway irrespective of additional HVEM-binding factors [30], [31]. Previous results, based both on DNA vaccines and adenovirus vectors, have demonstrated that the fusion of heterologous sequences at the C-terminus of gD target the protein to the outer face of the cytoplasmic membrane where the binding to HVEM occurs, leading to the enhanced activation of CD8^+^ T cell responses to the heterologous antigen [13], [15], [32], [36]. Similar but less significant results have also been obtained with recombinant purified proteins [14], further suggesting that endogenous expression and membrane localization contributes to the gD-mediated CD8^+^ T cell adjuvant effects.

Induction of cytotoxic CD8^+^ T cell responses represents an important immunological correlate for vaccines aiming therapeutic activation of protective immunity, such as those involved with eradication of chronic infections and cancer [47], [48]. Indeed, cytotoxic CD8^+^ T cells are involved in the resolution of infections with HIV [49]–[51], HSV [52], [53] and HPV-induced tumors [15], [27], [54]. The present study demonstrated that bicistronic DNA vaccines encoding gD fused antigens could enhance the induction of antigen-specific cytotoxic CD8^+^ T cell responses and confer protective immunity to virus infection and therapeutic control of tumor growth. These findings also indicate that a trivalent vaccine formulation targeting HIV, HSV and HPV associated cancer is a feasible goal and represent an interesting approach to be tested in the near future.
